# Phase-II Clinical Validation of a Powered Exoskeleton for the Treatment of Elbow Spasticity

**DOI:** 10.3389/fnins.2017.00261

**Published:** 2017-05-12

**Authors:** Simona Crea, Marco Cempini, Stefano Mazzoleni, Maria Chiara Carrozza, Federico Posteraro, Nicola Vitiello

**Affiliations:** ^1^The BioRobotics Institute, Scuola Superiore Sant'AnnaPisa, Italy; ^2^Shirley Ryan AbilityLabChicago, IL, USA; ^3^Feinberg School of Medicine, Northwestern UniversityChicago, IL, USA; ^4^Rehabilitation Bioengineering LaboratoryVolterra, Italy; ^5^Rehabilitation Department, Versilia Hospital, Azienda USL Toscana Nord OvestViareggio, Italy; ^6^Fondazione Don Carlo GnocchiFirenze, Italy

**Keywords:** robotics, rehabilitation, stroke, upper limb, exoskeleton, spasticity

## Abstract

**Introduction:** Spasticity is a typical motor disorder in patients affected by stroke. Typically post-stroke rehabilitation consists of repetition of mobilization exercises on impaired limbs, aimed to reduce muscle hypertonia and mitigate spastic reflexes. It is currently strongly debated if the treatment's effectiveness improves with the timeliness of its adoption; in particular, starting intensive rehabilitation as close as possible to the stroke event may counteract the growth and postpone the onset of spasticity. In this paper we present a phase-II clinical validation of a robotic exoskeleton in treating subacute post-stroke patients.

**Methods:** Seventeen post-stroke patients participated in 10 daily rehabilitation sessions using the NEUROExos Elbow Module exoskeleton, each one lasting 45 min: the exercises consisted of *isokinetic passive mobilization* of the elbow, *with torque threshold* to detect excessive user's resistance to the movement. We investigated the *safety* by reporting possible adverse events, such as mechanical, electrical or software failures of the device or injuries or pain experienced by the patient. As regards the *efficacy*, the Modified Ashworth Scale, was identified as primary outcome measure and the NEEM metrics describing elbow joint resistance to passive extension (i.e., maximum extension torque and zero-torque angle) as secondary outcomes.

**Results:** During the entire duration of the treatments no failures or adverse events for the patients were reported. No statistically significant differences were found in the Modified Ashworth Scale scores, between pre-treatment and post-treatment and between post-treatment and follow-up sessions, indicating the absence of spasticity increase throughout (14 days) and after (3–4 months follow-up) the treatment. Exoskeleton metrics confirmed the absence of significant difference in between pre- and post-treatment data, whereas intra-session data highlighted significant differences in the secondary outcomes, toward a decrease of the subject's joint resistance.

**Conclusions:** The results show that our robotic exoskeleton can be safely used for prolonged sessions in post-stroke and suggest that intensive early rehabilitation treatment may prevent the occurrence of spasticity at a later stage. Moreover, the NEEM metrics were found to be reliable compared to the Modified Ashworth Scale and sensitive to revealing intra-session changes of elbow resistance to passive extension, in agreement with clinical evidences.

## Introduction

Stroke has been recognized as one of the major causes of long-term movement disabilities in the elderly, and in the last years its contingency is continuously increasing (Feigin et al., 2003). A huge number of stroke-related disabilities dramatically influence the lives of stroke survivors, of which spasticity is a significant component (O'Brien et al., 1996). Clinically, spasticity is defined as the occurrence of increased involuntary resistance to passive movements. However, this phenomenon reflects a wider spectrum of clinical problems, including abnormal limb posture and hyperactive cutaneous and tendon reflexes. After stroke, the initial usual occurrence of flaccidity and hypotonia is followed by spasticity in about 20% of patients, after a time interval that is highly variable and may occur in the short-, medium-, or long-term post-stroke period depending on a large number of factors (Ward, 2012). Clinical studies have emphasized that preventing spasticity and treating emerging spasticity in a timely manner is essential to improve the neurologic and articular pattern of the impaired limb (Ottenbacher and Jannell, 1993).

Typical rehabilitation therapy for stroke survivors helps them relearn skills that are lost due to brain damage; in particular, patients are prompted to engage in passive or active range-of-motion exercises to strengthen their impaired limbs, as an integral part of daily management (Langhorne et al., 2011). Indeed, while they tend to avoid using paretic limbs, it has been widely demonstrated that their repetitive mobilization (either passive or active) can reduce muscle hypertonia, thus lowering joint spasticity and encouraging brain plasticity (Lum P. S. et al., 2002; Colombo et al., 2005; Masiero et al., 2007). Among the different rehabilitation exercises (e.g., passive, active-assisted, active as needed, active-resisted), the passive movement of the patient's joints has been demonstrated to maintain the range of motion (*ROM*) at the joints and flexibility in the muscles and connective tissue, as well as reduce muscle hypertonia and resistance to passive movement (Schmit et al., 2000; Lum P. et al., 2002; Nuyens et al., 2002). One of the key components of effective post-stroke rehabilitation is also the early mobilization of the paretic limb (Langhorne and Pollock, 2002).

When treating muscle contractures, the mobilization of contracted articulations involves close physical contact between the therapist and the patient: the therapist needs to regulate the strength and speed of the motion of the patient's articulations, in order to correct any compensatory strategies and comply with sudden spasticity. In this scenario, using robotic platforms that are capable of automating the repetitive motions usually performed by the physical therapist (Kwakkel et al., 2007) and of complying with sudden spasticity may facilitate high-intensity rehabilitation.

Many devices have been specifically developed for the neuro-rehabilitation of stroke patients, allowing patients to perform specific movements of the limbs. Extensive reviews of robotic devices for upper-limb neuro-rehabilitation have been carried out (Pizzi et al., 2005; Turchetti et al., 2014): many upper-limb robotic devices have been applied in stroke rehabilitation and their effectiveness has been evaluated at the start and end of treatment by means of widely used clinical assessment scales (Volpe et al., 2000; Masiero et al., 2007; Mazzoleni et al., 2013a,b) and sometimes even by measurements performed using robotic systems (Colombo et al., 2005; Mazzoleni et al., 2011).

Within the above framework, at The BioRobotics Institute of Scuola Superiore Sant'Anna we recently developed the NEUROExos, a powered elbow exoskeleton, designed to perform both passive and active elbow joint mobilization in post-stroke patients (Vitiello et al., 2013). A modified version of the device, the NEUROExos Elbow Module (NEEM) holds certification as a class-II medical device (compliant with EU regulations for medical devices, i.e., IEC EN 60601-1:2007 and EN ISO 14971:2012) and is approved for in-clinic use (Vitiello et al., 2016).

In this paper, a group of 17 post-stroke patients in their sub-acute phase were treated with the NEEM system on a daily basis, in addition to traditional physical therapy. The outcome was assessed in terms of *system safety* and *efficacy*. Safety was investigated by reporting possible adverse events, such as mechanical, electrical or software failures of the device or injuries or pain experienced by the patient. To test the treatment *efficacy*, the Modified Ashworth Scale (*MAS*, Ashworth, 1964; Bohannon and Smith, 1987; Pandyan et al., 1999) was identified as primary outcome measure and the NEEM metrics describing elbow joint resistance to passive extension (i.e., maximum extension torque and zero-torque angle) as secondary outcomes. We expected that intense therapy performed in the sub-acute phase following the stroke would be effective in preventing elbow spasticity from occurring at a later stage (i.e., 3–4 months after the stroke). Similar to other phase-II studies in the field of post-stroke rehabilitation (van Heugten et al., 1998), the present study included only one group of patients. A non-controlled group of study was essential to understand whether the proposed therapy could provide positive results, and was intended to be preparatory for a phase-III randomized control trial, where the efficacy of the proposed therapy will be compared with other conventional therapies. As a second objective, similarly to other studies with robot-assisted therapy (Colombo et al., 2005), the results from the robot metrics were validated against the outcomes from the *MAS* and clinical evidence from the state-of-the-art.

## Methods

### The NEEM system

**Figure 1:**
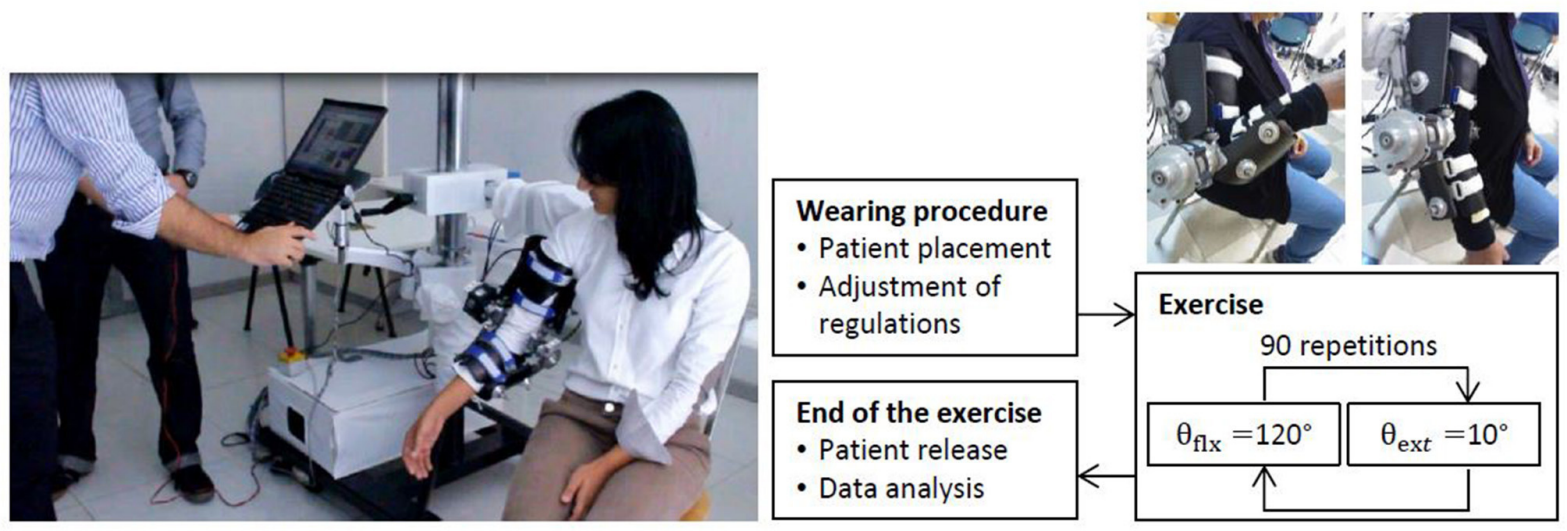
**Overview of the NEEM system in the clinical scenario**.

While a detailed description of the mechatronic modules of the NEEM platform, along with its performance, is given in Vitiello et al. (2016), for the sake of clarity we will summarize its main features below. The NEEM is a powered elbow exoskeleton designed to ensure maximum comfort and safety to the patient (Figure 1). It embodies many key design solutions for enhanced ergonomics. Firstly, a compact and light-weight mechanical structure, with double-shelled links and a wide physical human-robot interaction area, optimizes interaction comfort. Secondly, a four-degree-of-freedom passive mechanism relieves the elbow articulation from loads other than the actuated flexion/extension torque, and at the same time ensures appropriate alignment between human and robot joint axes. Thirdly, a series elastic actuation (SEA) unit (Giovacchini et al., 2014) allows compliant human-robot interaction as well as reliable measurement of the powered torque. The actuation unit is housed remotely in a box on the exoskeleton stand, in order to minimize the weight of the moving part. The actuation unit employs a 400 W DC brushless motor (EC motor EC60, Maxon Motor®, Sachseln, Switzerland), a Harmonic Drive reduction stage (transmission ratio 1:80), a torque limiter acting as safety clutch on the gear output, which disengages the actuation unit from the transmission means in case of torque values higher than a pre-defined threshold (set to 35 Nm in this study), and a grooved pulley. Torque is transmitted from this pulley to the exoskeleton actuated joint through a tendon-sheath system: two antagonist tendons, routed by flexible Bowden hoses, transmit rotation from the actuation unit pulley to the exoskeleton joint. A torsion spring is deployed between the actuated pulley and the exoskeleton. This element provides series elasticity, ensuring compliant interaction and accurate force transduction. Two 32-bit absolute encoders (RESOLUTE™, RESA30USA052B ring plus RA32BAA052B30F readhead, Renishaw®, Wotton-under-Edge, UK) sensorize the spring, one for each extremity. The encoder more proximal to the human joint (i.e., the joint position encoder) provides the elbow joint angular value, namely the *measured joint angle*, while the difference between the two encoder readings gives torsion spring deformation and, indirectly, the value of the transmitted torque. The wearable and moving part of the system (the powered orthosis) hangs from an adjustable spherical gimbal, which is mounted on an extensible horizontal cantilever bar supported by the main vertical stand. It allows manual adjustments to best fit the orthosis to the patient's body. Finally, the entire system was assembled onto a wheeled platform, in order to allow easy transportation within the clinical setting.

The NEEM control system is based on a two-layered hierarchical architecture. The NEEM low-level control implements two basic working modalities: (a) position control and (b) torque control. Under the position control mode, the robot joint is displaced along pre-defined angular trajectories (i.e., the *desired angle*) thanks to the action of a closed-loop proportional-integrative (PI) regulator acting on the error between the desired and measured joint angle. The output of the PI regulator is a desired current which is supplied to the DC motor by means of a commercial servo amplifier (EPOS2 70/10, Maxon Motor®, Sachseln, Switzerland). Under the torque control mode, a PI closed-loop compensator is used to control torsional spring deformation and so apply a desired torque to the user joint. Under this control mode, when the reference torque is null, the exoskeleton joint becomes “transparent” and the user can freely move.

The high-level control layer has the form of a finite-state machine, which implements a warm-up routine and a set of pre-defined rehabilitation exercises (i.e., passive, active-assisted, active-resisted, similar to those described in Prange et al., 2006). Once the system is powered on, the finite-state machine enters the so-called “Initialization” state: the exoskeleton holds the current angular position, under the position control mode action. At this point, the physical therapist (namely the experimenter) can switch the machine to the so-called “Wear” state: the system smoothly switches to the torque control mode with null torque as a desired reference, i.e., the exoskeleton joint is free to move. This way the therapist can help the patient wear the exoskeleton. Once the exoskeleton is fastened to the patient's limb, the therapist can select and start any of the desired rehabilitation exercises (Figure 1).

### Rehabilitation exercise

**Figure 2:**
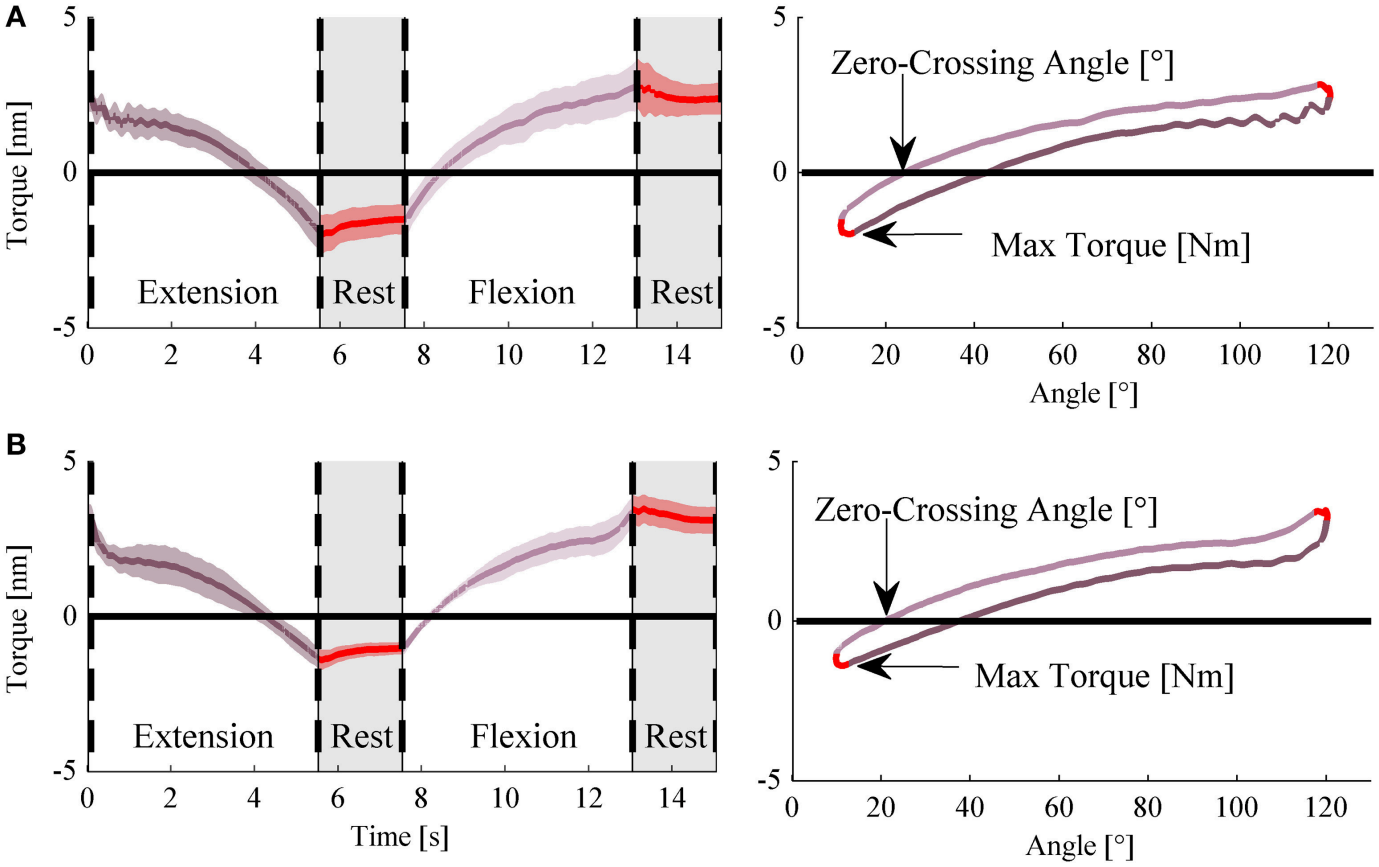
**Figure **1** NEEM measurement and indices for a representative subject. (A)** Average applied torque during a flexion/extension cycle, during the first treatment session (Day #1). **(B)** Average applied torque and measured angle during a flexion/extension exercise, during the last treatment session (Day #10).

The rehabilitation exercise that was selected for this work was the so-called *isokinetic passive mobilization with torque threshold*. The exoskeleton was programmed to displace –under the position control mode– the elbow joint of the patient along a predetermined flexion/extension trajectory (henceforth named “cycle”). In particular, the exoskeleton moved the elbow joint from an initial flexed configuration (i.e., θ_*flx*_ = 120°, θ being the patient's elbow angle measured by the exoskeleto) to a full-extended configuration (i.e., θ_*ext*_ = 10°) at a constant velocity. For safety purposes, threshold values for the maximum allowed torque, in flexion, $\tau_{flx}^{th}$, and extension, $\tau_{ext}^{th}$, applied by the exoskeleton were set for each subject: when the transmitted torque exceeded one of these thresholds, as in the case of any abnormal/spastic muscle activity, the system switched to the torque-control mode with the desired torque set to zero. This way, the robot could smoothly comply with the user's spontaneous movement and prevent spastic muscle strength from increasing, with the consequent worsening of stretch reflexes and hypertonia. Each flexion-extension cycle can be divided into four phases (Figure 2), namely: (i) “*elbow extension*,” the exoskeleton moved at a speed of 20°/s from θ_*flx*_ to θ_*ext*_; (ii) “*rest*,” the exoskeleton held the fully extended position for 2 s; (iii) “*elbow flexion*,” the exoskeleton moved at the same speed from θ_*ext*_ to θ_*flx*_; and (iv) “*rest*,” the exoskeleton kept the fully flexed position for 2 s. Exoskeleton speed was kept at 20°/s to reduce the probability to evoke stretch reflexes during execution of the movement (Levin et al., 2000). Speed, as well as the values of θ_*ext*_ and θ_*flx*_ were constant across the patients. The threshold values for the applied torque were set as follows. For the flexion (positive) torque, the threshold was equal for all patients and set to $\tau_{flx}^{th}=$ 7 N·m. For the extension (negative) torque, the threshold changed among subjects in the range $\tau_{ext}^{th}=$ −5 ± 1 N·m.

### Experimental protocol

The experimental protocol was approved by the local ethical committee (Azienda Ospedaliera Universitaria of Pisa, reference number 3919, year 2014) and carried out in accordance with the principles stated by the Declaration of Helsinki. The trial was also registered on ClinicalTrials.gov (Identifier is NCT02934646). All participants signed a written informed consent before starting the experiment. A physical therapist assessed the participants' elbow *ROM*, Chedoke-McMaster Stroke Assessment Measure (*CMSA*) (Gowland et al., 1993), and *MAS* before the beginning of the treatment. After the first assessment was completed, the subjects were seated next to the exoskeleton, on either an armless chair or their own wheelchair, as depicted in Figure 1. When using the wheelchair, the right armrest was removed for the entire duration of the exercise. A physical therapist helped the participant place his/her forearm on the lower link of the exoskeleton, and his/her upper arm on the upper link. The exoskeleton vertical and horizontal position and rotation on the main stand were adjusted to best adapt the patient's arm to the powered orthosis: the upper and lower back shells of the exoskeleton were adjusted so that they adhered completely to the limbs and so that the height of the patient's right shoulder was symmetrical to the left side. After adjusting the exoskeleton, the device was fastened to the user's arm by means of two belts. Subjects participated in 10 rehabilitation sessions, on consecutive working days, for 45 min, i.e., 90 flexion/extension cycles per session. When the subjects showed any discomfort in maintaining the seated position for 45 min, the duration of the treatment was set to 15 min. Subjects were allowed to skip up to two consecutive sessions. Subjects who skipped three consecutive sessions were excluded from the protocol. It is worth noting that after the first session, the subject-specific settings of the exoskeleton were not changed for the entire duration of treatment. After completing the last session, the subjects were evaluated once more by a physical therapist who quantified the post-treatment elbow *ROM* values and *MAS* scores. After 3 months from the stroke, the subjects were asked to participate in a follow-up screening to assess *MAS* and *ROM*.

The first assessment of the subject's *ROM* was used to set the limits on θ_*ext*_ and θ_*flx*_ and the torque threshold $\tau_{ext}^{th}$ so that the applied torque allowed to fully extend the patient's elbow without causing pain.

### Participants

Subjects enrolled in the study ranged in the 15th to the 35th day after stroke, at the beginning of the therapy. Plegia on the right upper limbs due to stroke was considered an inclusion criteria, while subjects with orthopedic pathologies at the elbow, e.g., articular blocks, were excluded from the study. Seventeen post-stroke subjects completed the protocol (aged 71 ± 14 years); 12 of them suffered from ischemic stroke, and the rest from hemorrhagic stroke. On average, subjects started the robot-assisted treatment in their subacute phase, i.e., the 25th day after the stroke (±5 days). Two additional subjects initially started the protocol but were then excluded since they skipped more than two sessions. Two out of 17 subjects performed treatment 15 min a day instead of 45 min because they could not maintain a sitting position for prolonged sessions. Out of the 17 participants, only 13 were able to attend the follow-up assessment.

All subjects fit into the maximum *ROM* of the NEEM, i.e., θ_*flx*_ = 120° and θ_*ext*_ = 10° and could perform the movement at the pre-defined constant speed of 20°/s.

### NEEM data collection and analysis

We collected the following variables from the exoskeleton sensing apparatus: the applied torque τ, and the elbow desired θ_*des*_ and measured θ angle. All variables were sampled and stored at 100 Hz. After completion of each session, data were downloaded from the NEEM control unit and stored for offline data analysis. We extracted the following metrics from the variables of each flexion-extension cycle, as indicators of the *resistance to the elbow extension* (Figure 2):

- Maximum Extension Torque (*MET*): the torque exerted by the NEEM at the fully extended position;
- Zero-Torque Angle (*ZTA*): the angle at which the applied torque was zero during the *elbow extension* phase of the cycle.

Notably, the corresponding flexion parameters were not considered in this study as all the participants had spasticity on the elbow flexors, thus the resistance to elbow extension is more informative about the status of the spasticity.

### Statistical analysis

The overall effect of the robot-mediated treatment was quantified using two approaches.

1. *MAS*. Friedman repeated measures analysis of variance on ranks was performed to detect the overall effects of the treatment on the *MAS* scores, in pre-treatment, post-treatment and follow-up data. *Post-hoc* pairwise multiple comparison of the *MAS* scores was performed using the Tukey test (i.e., pre-treatment with post-treatment data, post-treatment with follow-up data and pre-treatment with follow-up data).
2. NEEM metrics. The metrics extracted from the initial and final rehabilitative sessions were compared as explained hereafter. For each session, *MET* and *ZTA* were considered on the last 20 flexion/extension cycles (corresponding to the last 10 min of the trial). Data from the first and last 2 days of the treatment were grouped and median values extracted for each subject, to respectively compute representative values of the two initial sessions (i.e., *MET*_*Init Session*_, *ZTA*_*Init Session*_) and two final sessions (i.e., *MET*_*Final Session*_ and *ZTA*_*Final Session*_). Across-subject statistical analysis was performed separately for *MET* and *ZTA*, using a Wilcoxon signed-rank test.

In addition, we analyzed the NEEM metrics to assess the intra-session effect of the treatment, by comparing the initial and final cycles of each session. For each session, median values of the first and last 5 flexion/extension cycles were extracted. The median values of all the indices extracted from the initial and final cycles of each session (from Days 1 to 10) were computed. *MET* and *ZTA* data from all participants were thus grouped in four vectors, namely *MET*_*Init Cycle*_, *MET*_*Final Cycle*_, *ZTA*_*Init Cycle*_, and *ZTA*_*Final Cycle*_. A Wilcoxon signed-rank test was used for comparison of initial and final cycles values.

Statistical significance was set at *p* < 0.05. SigmaStat software (version 3.5) (Systat Software, San Jose, CA, USA) was used for statistical analysis.

## Results

### Safety

All participants easily wore the exoskeleton without reporting any discomfort. Subjects had different weights, arm sizes and upper-arm circumferences, so different inner-shell sizes were used to provide a comfortable wearability of the exoskeleton. Moreover, subjects had different levels of upper-limb motor impairments (CMSA scores ranging from 1 to 6). In addition, some of them could walk easily and sit on a normal armless chair while most of them needed to sit on their own wheelchair. All participants could easily hold a correct sitting posture next to the exoskeleton to effectively complete the rehabilitation sessions.

The device was tested for a total of more than 120 h. During the entire duration of the treatment no mechanical or electrical failures or reliability-related issues were reported; the clinical validation was completed without any damages to the device (or any of its components) or any undesired effects on the patients.

### Efficacy of the treatment

#### Overall assessment based on MAS scores

Table 1 reports the *MAS* scores and the *ROM* values for all the participants assessed in the pre-treatment, post-treatment and follow-up sessions. The *CMSA* scores are reported only for the pre-treatment evaluation. Friedman test revealed no changes in the *MAS* scores in pre-treatment, post-treatment and follow-up session [χ^2^(2, 17) = 0.5, *p* = 0.779]^1^, thus *post-hoc* pairwise comparison was not performed.

**Table 1:** **MAS scores and ROM values for each subject, assessed by physical therapists before and after the rehabilitation therapy with NEUROExos**.

**Subject [#]**	**1**	**2**	**3**	**4**	**5**	**6**	**7**	**8**	**9**	**10**	**11**	**12**	**13**	**14**	**15**	**16**	**17**
CMSA	6	5	5	6	6	2	6	6	6	6	6	6	6	1	2	2	1
Initial MAS	0	1	0	0	1	1	1	0	0	0	0	0	0	0	1	1	0
Initial ROM	150	150	150	115	150	150	150	150	150	150	150	150	150	150	150	150	150
Final MAS	0	1	0	0	1	1+	1	0	0	0	0	0	0	0	1	0	0
Final ROM	150	150	150	115	150	150	150	150	150	150	150	150	150	150	150	150	150
Follow-up MAS	0	1	0	0	1	1+	0	0	0	0	0	NA	NA	NA	0	1	NA
Follow-up ROM	150	150	150	115	150	150	150	150	150	150	150	NA	NA	NA	150	150	NA

*NA, Not Available*.

At the beginning of the treatment all patients showed a MAS score 0 or 1 and a full elbow ROM as they were recruited during the subacute phase when spasticity is not usually arisen.

Fifteen out of 17 participants presented the same value of *MAS* score and *ROM* in pre- and post-treatment. A decrease of the *MAS* score from 1 to 0 was observed in one subject (#16), and in another (#6) an increase from 1 to 1+. Ten out of 13 subjects who participated in the follow-up, showed the same *MAS* score in the follow-up and the post-treatment sessions; two subjects (#7 and #15) showed a decrease from 1 to 0, whereas an increase from 0 to 1 was observed in one subject (#16). In the follow-up session, one participant revealed moderate spasticity (*MAS* = 1+).

#### Overall assessment based on NEEM metrics

**Figure 3:**
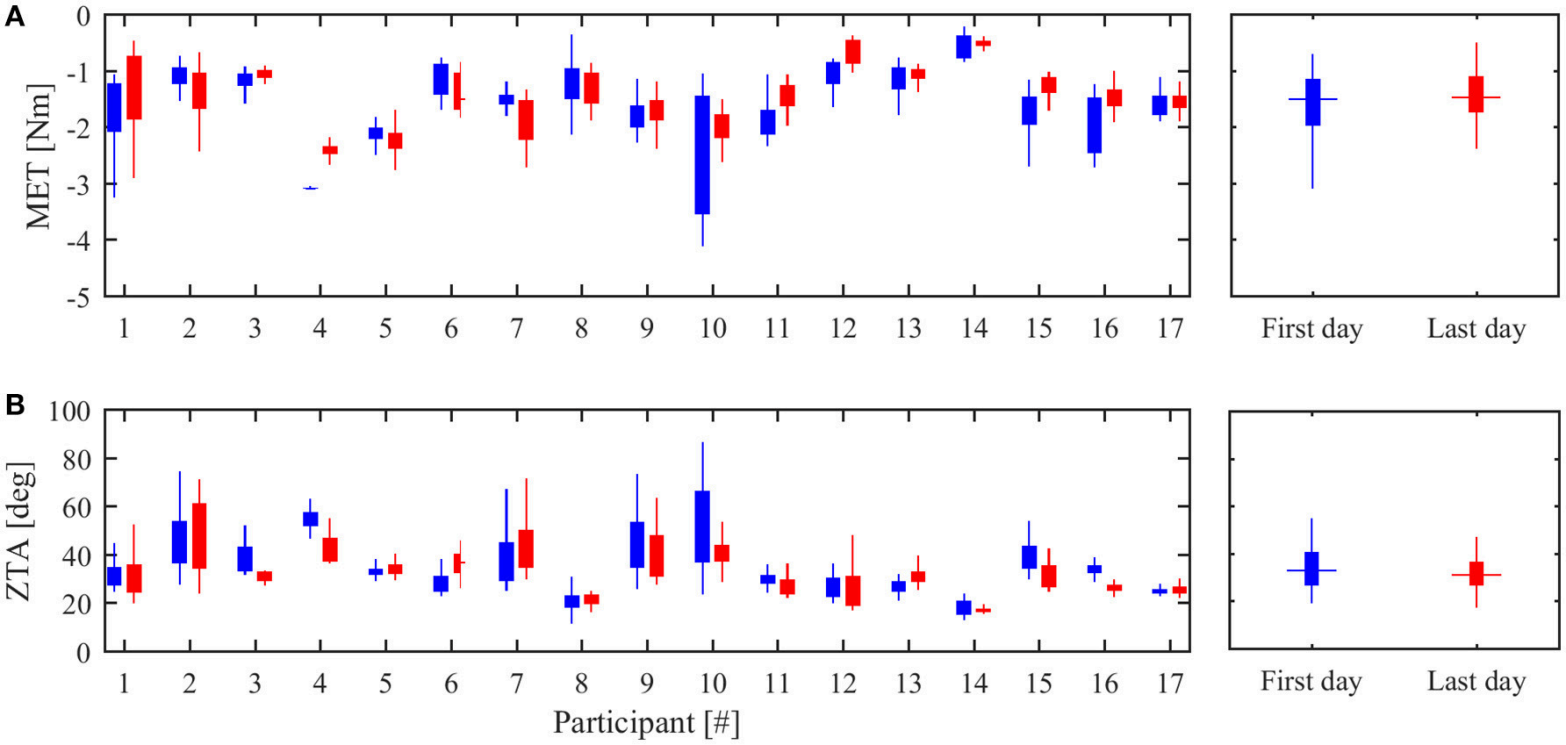
**Overall therapy effectiveness. (A)** Maximum extension torque (*MET*) in the first (blue) and last (red) day of treatment. **(B)** Zero-Torque Angle (*ZTA*) in the first (blue) and last (red) day of treatment. The left panels show the median values and 90% confidence interval for each subject. The right panel reports the inter-subject median value and confidence interval.

Figure 3 reports the results of the two indices extracted from the NEEM measurements, *MET* and *ZTA*. Data are presented both for each subject and as aggregated form. Comparison between the initial and final treatment sessions did not show any statistical difference in *MET* (*p* = 0.09) and *ZTA* (*p* = 0.28), in accordance with the *MAS* results. Table S1 (in Supplementary Materials) reports the numerical data.

**Figure 4:**
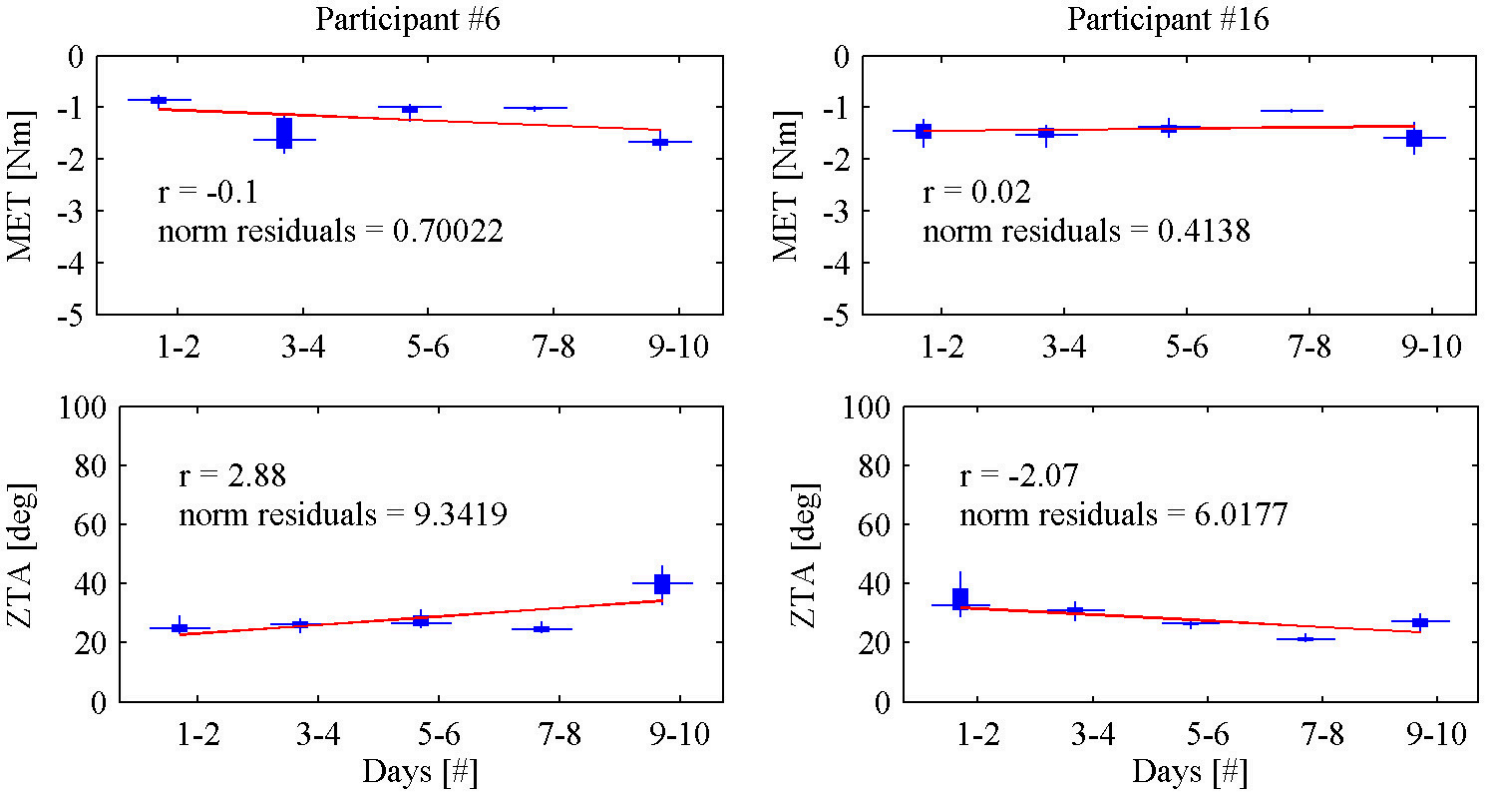
**Maximum extension torque (***MET***) and Zero-Torque Angle (***ZAT***) for Participant #6 and Participant #16 over all the treatment sessions**.

Subject #6, who presented an increased *MAS* score between pre- and post-treatment, also showed an increased *MET* (+27%, absolute value) and an increased *ZTA* (+28%); subject #16, who showed a decreased *MAS* score in the post-treatment, presented a decreased *MET* (−27%, absolute value) and a decreased *ZTA* (−25%). Figure 4 shows *MET* and *ZTA* values from subject #6 and subject #16 over all the treatment sessions. The regression line is calculated using the least-square technique and confirms the trend over the whole treatment.

### Intra-session treatment assessment based on NEEM metrics

**Figure 5:**
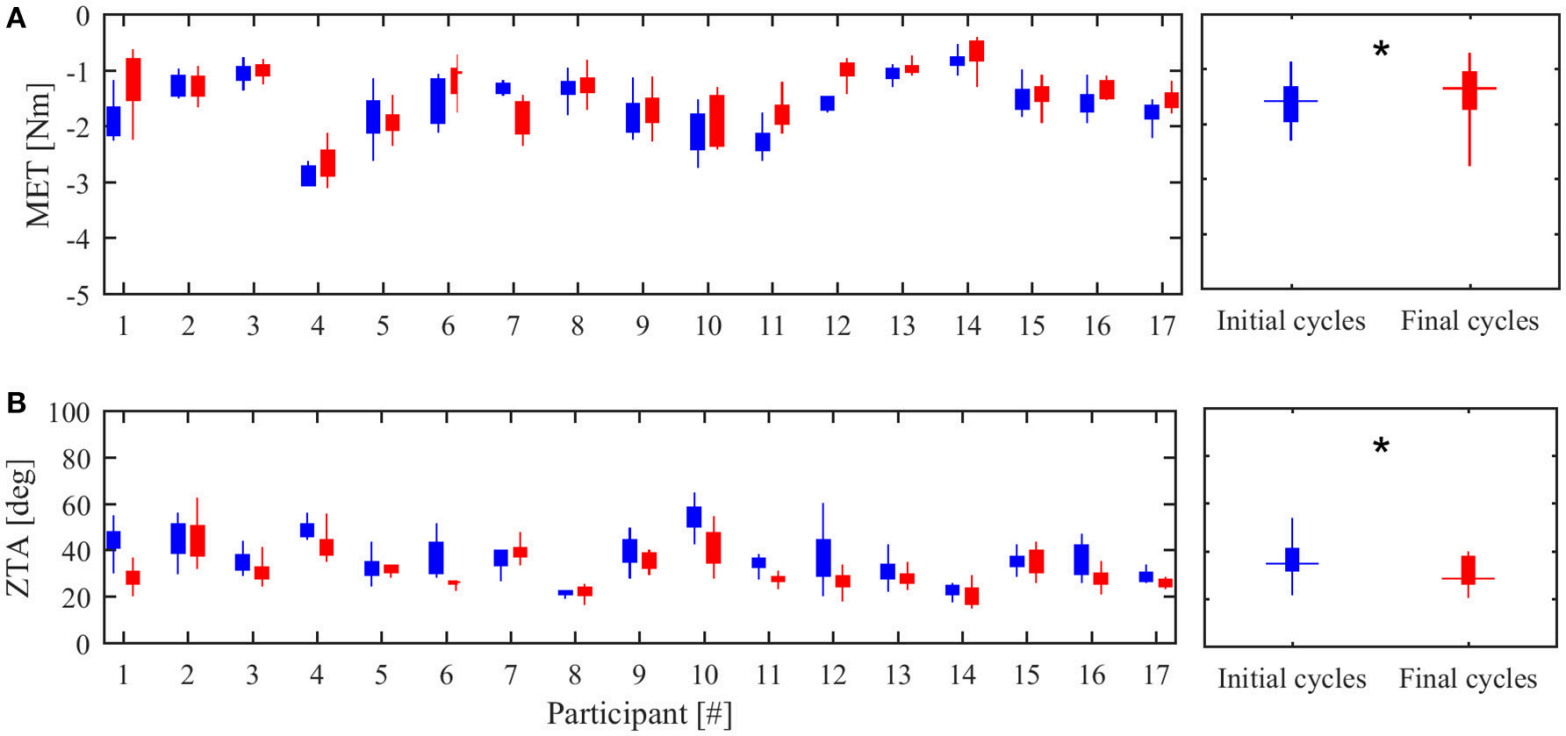
**Intra-day therapy effectiveness. (A)** Maximum extension torque (*MET*) in the initial (blue) and final (red) cycles of treatment. **(B)** Zero-Torque Angle (*ZTA*) in the initial (blue) and final (red) cycles of treatment. The left panels show the median values and 90% confidence interval for each subject. The right panel reports the inter-subject median value and confidence interval. ^*^indicates statistical significant difference (*p* < 0.05).

Figure 5 shows the comparison between *MET* and *ZTA* values referred to the first and last cycles of the rehabilitative sessions. Data are presented both for each participant and as aggregated form. Comparison between the initial and final values revealed a statistical difference in *MET* (*p* = 0.020) and *ZTA* (*p* = 0.003).

In 10 subjects, when comparing the initial and final cycles of the treatment sessions, both *MET* and *ZTA* values substantially decreased (at least −10% from their initial values on both parameters). One subject (#7) showed increased *MET* and *ZTA* values (over +10% on both parameters), whereas the others showed variations in the range of ±10% of the initial values (#3, #4, #5, #8, #13, #14).

## Discussion

### Overall usability and safety

The use of robotic devices for rehabilitation can provide numerous advantages in terms of intensity and repeatability of the treatment for the patient and decrease of physical effort for the physiotherapists (Turchetti et al., 2014). Moreover, robot-assisted rehabilitation treatments may provide better optimization of healthcare resources (e.g., therapists can supervise more than one patient at a time, without reducing the effort dedicated to each patient). In addition robotic devices are able to provide quantitative evaluation of motor recovery by means of specific parameters that can characterize the rate of improvement of the patients (Colombo et al., 2005). However, for the treatment of upper-limb spasticity, studies using the MIT-Manus (Krebs et al., 2004), Bi-Manu Track (Hesse et al., 2003), and REHAROB (Fazekas et al., 2007) robots proved that passive repetitive exercises could improve spasticity in chronic stroke patients, but few of them correlated the treatments with the rising of spasticity at a later stage in sub-acute patients. Moreover, just recently it has been demonstrated that from a functional perspective, there is significant effect in favor of robot-assisted therapy with respect to traditional therapies (Staubli et al., 2009; Mehrholz et al., 2012), whereas previous review studies could not prove it (Prange et al., 2006; Kwakkel et al., 2007).

Subjects having different levels of motor impairments were able to complete the rehabilitation sessions characterized by the same flexion and extension angle and speed values.

This study shows the safety of the device for treating patients with different body sizes and motor impairments, and for prolonged sessions.

Moreover, it contributes to the investigation of efficacy-related aspects in robotic post-stroke rehabilitation.

### Efficacy of the therapy: early mobilization of the paretic limb could contribute to reduced risk of developing spasticity

Although a growing number of studies evaluated the prevalence of spasticity among the post-stroke population, the huge variability in time of onset of spasticity still impedes the determination of a universal prevalence rate (Ward, 2012).

One of the most recent studies on this topic reported that within 2 weeks after stroke, 24.5% of patients develop increased muscle tone (i.e., *MAS* ≥ 1), while after 16 weeks spasticity increases only slightly, up to 26.7%. Results highlight that in 98% of the patients who developed spasticity within the first 2 weeks, this is still present after 16 weeks (Wissel et al., 2010). Another study reports a similar finding: patients who develop spasticity after 3 months from stroke (i.e., 19% of patients), in general continue to report spasticity one and a half years later (Sommerfeld et al., 2004). At the same time, some patients stopped reporting spasticity, even though this outcome is rather rare. In line with the above mentioned studies (Sommerfeld et al., 2004; Wissel et al., 2010), in our population all the patients, but one, reporting spasticity in the pre-evaluation session (i.e., *MAS* ≥ 1), reported the same after treatment and in the follow-up.

Recently, Kong and colleagues (Kong et al., 2012) reported that 3 months after stroke, 33% of patients present spasticity, 65% of which moderate to severe spasticity (i.e., *MAS* ≥ 1+). Strikingly, our results show that among the 13 subjects who reported spasticity in the follow-up (*MAS* >0), only one participant showed moderate spasticity (with *MAS* = 1+), and none reported severe spasticity (i.e., *MAS* > 1+). The possible reasons to explain this positive outcome can be two-fold.

On the one hand the spasticity treatment we provided through the NEEM in the early post-stroke phase, prior to the progression of moderate or severe spasticity (Paolucci et al., 2000; Leathley et al., 2004), could effectively prevent the rising of spasticity in the later stage. This hypothesis is grounded on results reported by several studies. In particular, O'Dwyer and colleagues examined 24 hemiparetic patients recruited within 13 months of stroke and demonstrated how preventing or treating contractures in the early rehabilitation phase, is paramount to reduce the occurrence of spasticity (O'Dwyer et al., 1996). The intense rehabilitation treatment provided in this study by means of the NEEM can doubtfully be replicated by physical therapists, due to the huge amount of time and effort, and such intense early mobilization could have contributed to preventing the rising of severe spasticity in the following months.

On the other hand, it might be possible that prevalence rates calculated on our relatively-small group of patients, despite being similar to many similar studies on robot-assisted robotic treatment, can be slightly different from the statistics reported by the studies carried out on larger populations. It is worth noting that in this study, the absence of a control group prevents us from drawing conclusions about the *relative efficacy* of robot-assisted therapy with respect to other conventional physical therapies or spontaneous improvements: in the future, a randomized control trial will be necessary to evaluate the effects of different rehabilitation approaches to treating emerging spasticity.

### NEEM metrics could reliably assess the outcome of the rehabilitation

NEEM metrics has been validated against two benchmarks, i.e., the *MAS* scores and evidence from the state-of-the-art.

First of all, NEEM metrics was used to assess the overall treatment effects and outcome compared to the *MAS* scores. In agreement with results obtained from the *MAS* scores, both *MET* and *ZTA* values showed no statistical differences between pre- and post-treatment values. The qualitative comparison between device measurements and traditional clinical scales revealed that the robot measurements could detect changes in patients who reported a change in the *MAS* score (see subject #6 and #16). At the same time, the device revealed to be more sensitive in detecting small changes in joint resistance to passive extension since some participants who did not reported any *MAS* changes from pre- to post-treatment, showed visible differences in *MET* and *ZTA*.

Second, we validated intra-session results from NEEM metrics with evidence from the state of the art. It has been widely demonstrated that within a rehabilitation session with passive mobilization of the joint, its resistance to extension decreases (Nuyens et al., 2002). Indeed, muscle stretching and joint mobilization is a common intervention for treatment of spastic hypertonia or muscle contractures, which is based on the clinical experience that resistance decreases during repeated passive movements. Thus, we expected this result in almost all the participants and NEEM indices confirmed a statistical reduction of the elbow resistance, highlighting at the same time the reliability of the measurements extracted from the variable collected by the device.

The benchmark with the *MAS* scores, that is a golden standard for the assessment of joint spasticity, and evidences from the state of the art proved that the robot measurements could reliably describe the overall outcome of a rehabilitation treatment and suggests the possible use of the device not only for providing the treatment, but also as a tool to assess the outcome of a therapeutic treatment in an objective manner.

## Conclusions

The results of this study proved the usability and safety of the NEEM in rehabilitation; moreover, the results suggested that intense early rehabilitation could contribute to preventing elbow spasticity from occurring at a later stage (3–4 months after stroke); however the small sample size does not allow deriving straightforward conclusions applicable to larger populations.

The metrics extracted from the robot measurements were consistent with the MAS scale suggesting that the device could be potentially used as a tool to assess the outcome of rehabilitation. Notably, other features can be extracted from the NEEM measures (e.g., positive/negative delivered energy or power, active ROM, zero-torque angle and maximum torque in the flexion phase) and it can be easily synchronized with commercial electromiographic devices and allow measuring muscle activity.

The positive findings of this work invite further investigations in order to test the effectiveness of the proposed approach in a large-scale clinical study, where different therapy modalities, such as active-assisted or active-resisted exercises, can be proved to be able to prevent the occurrence of spasticity.

## Author contributions

Study concept and design: SC, MC, NV, MCC, FP. Acquisition of data: SC. Statistical analysis and interpretation of data: SC, MC, NV, FP, SM, MCC. Drafting of manuscript: SC, MC, NV, FP, SM, MCC. Study supervision: NV, FP.

## Funding

This study was partly supported by: the European Commission under the H2020 AIDE project (GA 645322) and by Regione Toscana under the RONDA project (Bando PAR FAS 2007–2013).

### Conflict of interest statement

The authors gdeclare that the research was conducted in the absence of any commercial or financial relationships that could be construed as a potential conflict of interest. SC, MCC, and NV have interests in the commercial exploitation of the patent application protecting the design of the NEEM torsional spring.

## Footnotes

^1^The software is able to consider missing data in the computation of statistical tests

## Supplementary material

Table S1 **Absolute value of MET and ZTA values for each subject**. Medians and 50% confidence intervals are reported.
