# Supplementary material for: Phase-II Clinical Validation of a Powered Exoskeleton for the Treatment of Elbow Spasticity
Source: Front Neurosci. 2017 May 12;11:261. doi: 10.3389/fnins.2017.00261 (PMC5427118; doi:10.3389/fnins.2017.00261)
Supplement: Table S1 — Absolute value of MET and ZTA values for each subject. Medians and 50% confidence intervals are reported. [file Table1.docx]

# Supplementary material

| Subject [#] | | 1 | 2 | 3 | 4 | 5 | 6 | 7 | 8 | 9 | 10 | 11 | 12 | 13 | 14 | 15 | 16 | 17 |
| --- | --- | --- | --- | --- | --- | --- | --- | --- | --- | --- | --- | --- | --- | --- | --- | --- | --- | --- |
| $\vert MET\vert$ | Day1 | 1.43 2.07-1.21 | 1.08  1.23-0.93 | 1.16 1.26-1.04 | 3.08 3.08-3.06 | 2.08 2.21-1.99 | 1.17 1.42-0.87 | 1.50 1.59-1.42 | 1.21  1.49-0.95 | 1.88 1.99-1.60 | 2.02 3.54-1.43 | 1.94 2.12-1.68 | 0.97  1.23-0.84 | 1.04 1.33-0.93 | 0.69 0.76-0.35 | 1.70 1.95-1.44 | 2.01 2.45-1.47 | 1.53  1.78-1.43 |
|  | Day10 | 1.09 1.85-0.73 | 1.27  1.67-1.02 | 1.05  1.12 0.98 | 2.37  2.46-2.32 | 2.26 2.38-2.09 | 1.49  1.68-1.03 | 1.85  2.22-1.51 | 1.17  1.58-1.02 | 1.69  1.87-1.50 | 2.07  2.19-1.76 | 1.51  1.62-1.24 | 0.60 0.86-0.43 | 1.01  1.14-0.96 | 0.49  0.55-0.45 | 1.14  1.39-1.11 | 1.46  1.62-1.31 | 1.53  1.65-1.43 |
| $ZTA$ | Day1 | 30.0  27.3-35.1 | 42.3  36.8-54.2 | 40.9 33.4-43.7 | 55.2  52.0-57.8 | 33.3 31.8-34.6 | 28.9 24.8-31.4 | 34.9 29.2-45.2 | 20.3 18.2-23.4 | 41.2 34.8-53.9 | 51.4 37.1-66.6 | 29.7 28.2-31.8 | 26.5 22.6-30.7 | 26.8 25.0-29.3 | 19.5 15.3-21.1 | 39.5 34.3-43.9 | 34.6 32.6-36.1 | 24.9 24.3-26.1 |
|  | Day10 | 31.0 24.6-36.2 | 47.6 34.6-61.4 | 31.6 29.3-33.2 | 39.8 37.2-47.2 | 35.0 32.4-36.3 | 37.1 32.6-40.8 | 36.8 34.7-50.6 | 20.9 19.9-23.6 | 35.1 31.2-48.3 | 41.5 37.3-44.2 | 28.0 23.9-30.2 | 27.4 19.0-31.5 | 30.4 28.9-33.5 | 17.4 16.5-17.9 | 30.3 26.6-35.7 | 26.6 25.4-27.7 | 25.3 24.3-27.2 |
